# Stimulation of Akt Phosphorylation and Glucose Transport by Metalloporphyrins with Peroxynitrite Decomposition Catalytic Activity

**DOI:** 10.3390/catal12080849

**Published:** 2022-08-02

**Authors:** Amanda M. Eccardt, Ross J. Pelzel, Thomas P. Bell, Jonathan S. Fisher

**Affiliations:** 1 Department of Biology, Saint Louis University, St. Louis, MO, USA; 2 Pfizer Inc., Chesterfield, MO, USA; 3 Graduate Program in Neuroscience, University of Minnesota, Minneapolis, MN, USA; 4 School of Medicine, Saint Louis University, St. Louis, MO, USA

**Keywords:** metalloporphyrins, peroxynitrite, insulin signaling

## Abstract

Iron porphyrin molecules such as hemin and iron(III) 4,4′,4″,4‴-(porphine-5,10,15,20-tetrayl)tetrakis(benzoic acid) (FeTBAP) have previously been shown to influence insulin signaling and glucose metabolism. We undertook this study to determine whether a catalytic action of iron porphyrin compounds would be related to their stimulation of insulin signaling and glucose uptake in C2C12 myotubes. FeTBAP did not display nitrite reductase activity or alter protein S-nitrosylation in myotubes, eliminating this as a candidate mode by which FeTBAP could act. FeTBAP displayed peroxynitrite decomposition catalytic activity in vitro. Additionally, in myotubes FeTBAP decreased protein nitration. The peroxynitrite decomposition catalyst Fe(III)5,10,15,20-tetrakis(4-sulfonatophenyl)porphyrinato chloride (FeTPPS) also decreased protein nitration in myotubes, but the iron porphyrin Fe(III)tetrakis(1-methyl-4-pyridyl)porphyrin pentachlorideporphyrin pentachloride (FeTMPyP) did not. FeTBAP and FeTPPS, but not FeTMPyP, showed in vitro peroxidase activity. Further, FeTBAP and FeTPPs, but not FeTMPyP, increased Akt phosphorylation and stimulated glucose uptake in myotubes. These findings suggest that iron porphyrin compounds with both peroxynitrite decomposition activity and peroxidase activity can stimulate insulin signaling and glucose transport in skeletal muscle cells.

## 1. Introduction

Biological and synthetically derived metalloporphyrins play roles in generation or scavenging of reactive nitrogen species (RNS). For example, it has been established that deoxymyoglobin has the ability to act as a nitrite reductase and thus catalyze the reduction of nitrite (NO_2_^−^) to form nitric oxide (NO) [1–3]. NO is a gaseous free radical that takes on a key role as a signaling molecule regulating physiological functions while also contributing to pathological progressions in skeletal muscle [4–7].

Peroxynitrite is a reactive nitrogen species (RNS) formed from the reaction between superoxide and nitric oxide [8]. Various iron-containing porphyrin compounds such as Fe(III)5,10,15,20-tetrakis(4-sulfonatophenyl)porphyrinato chloride (FeTPPS) and Fe(III)tetrakis(1-methyl-4-pyridyl)porphyrin pentachlorideporphyrin pentachloride (FeTMPyP) have been characterized as peroxynitrite decomposition catalysts due to their ability to catalytically scavenge peroxynitrite [9,10]. Peroxynitrite acts as a mediator of protein oxidation and nitration and can contribute to forms of metabolic dysfunction including insulin resistance [10–12].

Both NO and peroxynitrite have been shown to impair insulin signaling in skeletal muscle [4,5,12,13]. Nitric oxide increases S-nitrosylation of proteins due to its ability to react with cysteine residues [14,15]. This posttranslational modification prevents normal tyrosine phosphorylation of insulin receptor β with downstream suppression of phosphorylation of insulin receptor substrate 1 (IRS-1). Injection of mice with the peroxynitrite donor 3-morpholinosydnonimine (SIN-1) increased nitration of skeletal muscle IRS-1 and AKT [12]. This increase in nitrotyrosine was concomitant with decreased insulin signaling at the level of phosphatidylinositol 3-kinase activity and Akt phosphorylation [12].

We have recently shown that the metalloporphyrin iron(III) 4,4′,4″,4‴-(porphine-5,10,15,20-tetrayl)tetrakis(benzoic acid) (FeTBAP) could stimulate insulin signaling and glucose transport in skeletal muscle [16]. We attributed this to the peroxidase activity of FeTBAP. However, the stimulation of insulin signaling by FeTBAP occurred without a decrease in intracellular peroxide levels [16]. This suggests that some other action of FeTBAP mediates its effects on insulin signaling. Thus, we hypothesized that nitrite reductase or peroxynitrite decomposition activity of FeTBAP would be associated with the effects of FeTBAP on insulin signaling.

Here, we show that FeTBAP is a peroxynitrite decomposition catalyst and that it decreases protein nitration. We also show that FeTBAP and FeTPPS, which have both peroxidase and peroxynitrite decomposition activities, stimulate Akt phosphorylation and glucose transport in myotubes. These findings suggest potential roles of metallophorphryins with combined peroxidase and peroxynitrite decomposition activities in stimulation of insulin signaling and glucose uptake in skeletal muscle.

## 2. Results

### 2.1. FeTBAP Does Not Act as a Nitrite Reductase

**Figure 1.**
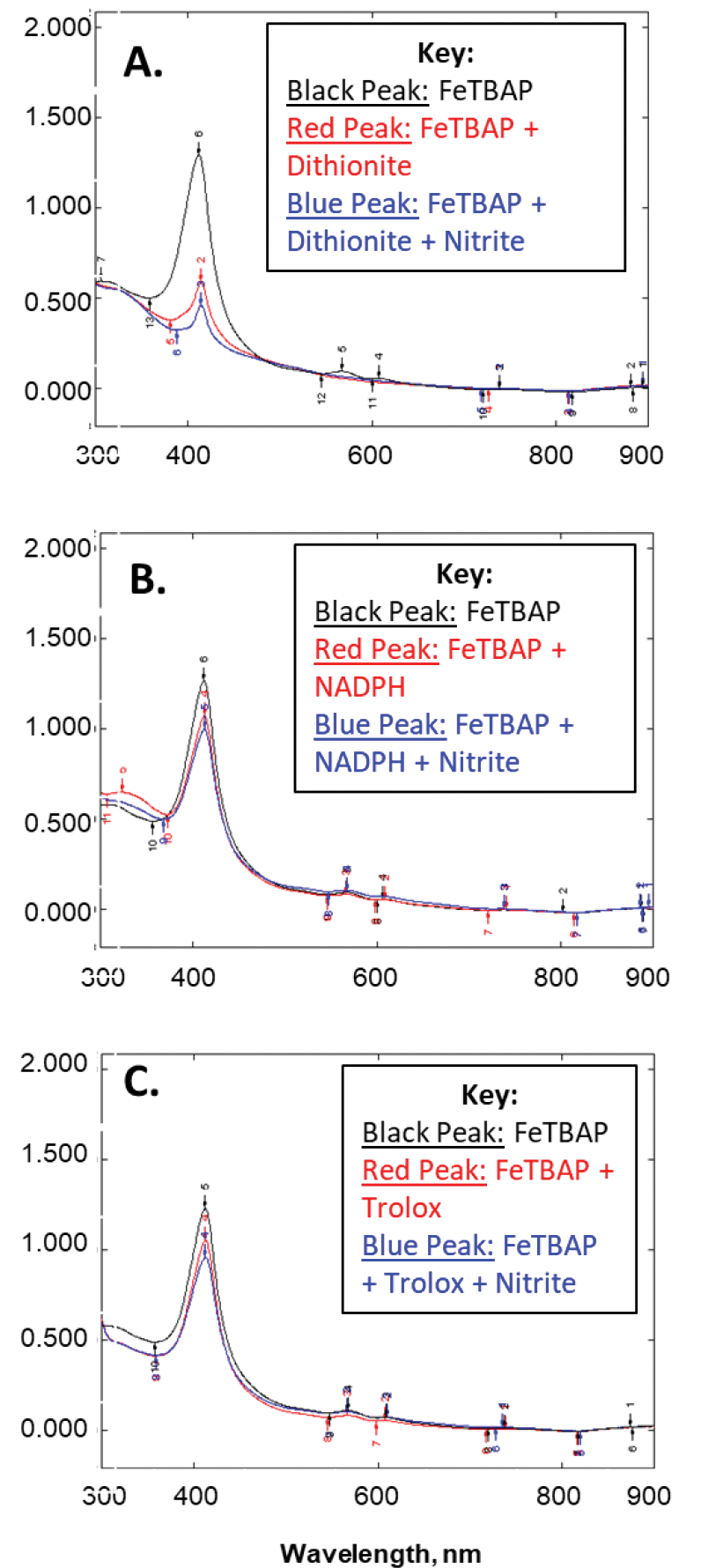
Decreased Soret band absorbance upon reduction is not reversed by nitrite. Absorbance spectra were obtained for 20 μM FeTBAP (black), FeTBAP after addition of reducing compounds (**A**. 100 μM dithionite, **B**. 100 μM NADPH, **C**. 100 μM Trolox; red), and after addition of 200 μM nitrite to reduced FeTBAP (blue).

Given the similarity in structure of FeTBAP to the heme group of myoglobin and the reports of the capabilities of deoxymyoglobin as a nitrite reductase [1–3], this study aimed at elucidating whether FeTBAP had similar function. Absorbance spectra monitoring the Soret band of FeTBAP illustrated a decrease in peak intensity after addition of reducing agents: dithionite, NADPH, or Trolox (Figure 1). Additionally, there was a further decrease in intensity of the Soret band upon the addition of nitrite (Figure 1). To corroborate these findings, S-nitrosylation was monitored in C2C12 myotubes. If FeTBAP acted as a nitrite reductase, this would result in nitric oxide production and thus an increase in S-nitrosylation [2,17,18]. However, after treatment with FeTBAP there was no difference in S-nitrosylation versus the control (data not shown). This suggests that under our conditions FeTBAP does not act as a nitrite reductase.

### 2.2. FeTBAP Acts as a Peroxynitrite Decomposition Catalyst

**Figure 2.**
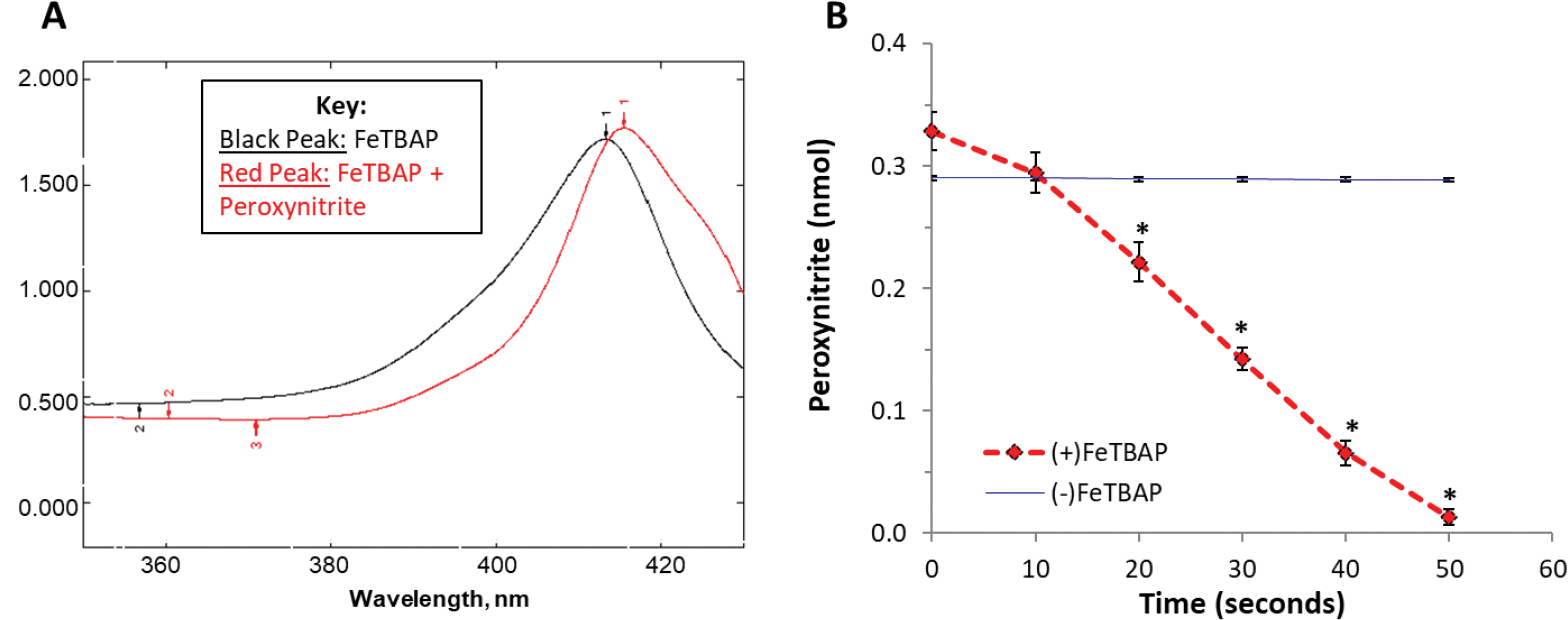
FeTBAP acts as a peroxynitrite decomposition catalyst. (**A**) Absorbance spectra of 20 μM FeTBAP before (black curve) and after (red curve) the addition of 300 μM peroxynitrite. (**B**) Peroxynitrite composition monitored at 302 nm for 20 μM FeTBAP. * *p* < 0.05 versus control without FeTBAP, n = 3–4/group.

Next, we examined a role of FeTBAP in relation to peroxynitrite. Previous studies have shown that iron porphyrins such as FeTMPyP and FeTPPS act as peroxynitrite decomposition catalysts [9,10]. As shownin Figure 2A, incubation with peroxynitrite causes a shift and an increase in peak intensity of the FeTBAP Soret band. This spectral shift and increase in peak intensity is consistent with previous findings in studies of Fe-porphyrins as peroxynitrite decomposition catalysts [9]. Decomposition of peroxynitrite was then monitored at 302 nm. Peroxynitrite rapidly decomposed in the presence of FeTBAP (Figure 2B, * *p* < 0.05 versus (−) FeTBAP). Taken together, these data suggest that FeTBAP is a peroxynitrite decomposition catalyst.

### 2.3. FeTBAP Decreases Nitration of Tyrosine Residues

**Figure 3.**
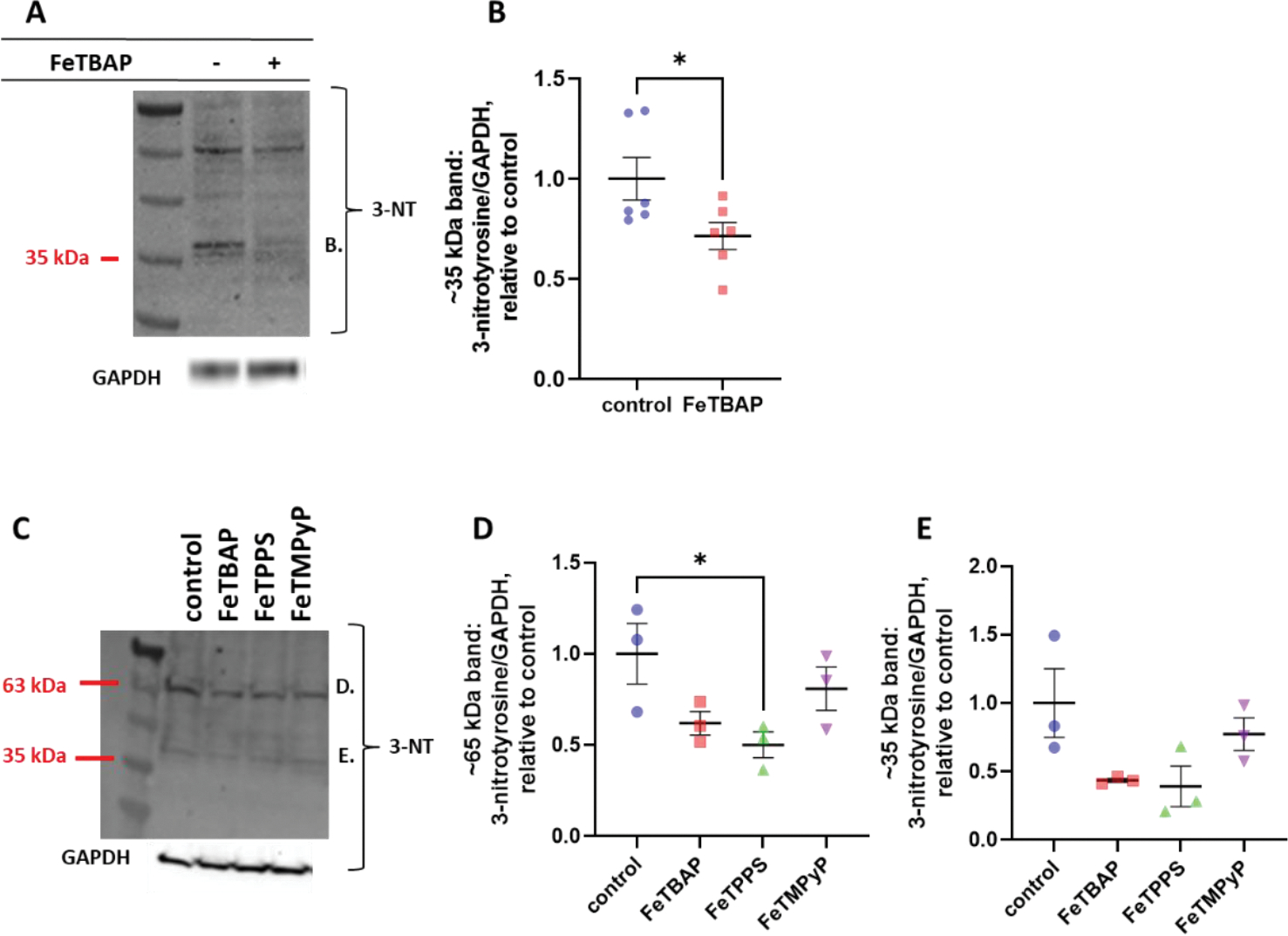
FeTBAP and FeTPPS decrease nitrotyrosine levels in C2C12 myotubes. C2C12 myotubes were incubated in the absence or presence of 150 μM FeTBAP, FeTPPS, or FeTMPyP for 18 h. (**A**,**B**) Western blot and quantitation for 3-nitrotyrosine and glyceraldehyde phosphate dehydrogenase (GAPDH) after incubation with FeTBAP. (**C**-**E**) Western blot and quantitation for 3-nitrotyrosine and GAPDH after incubation with FeTBAP, FeTPPS, or FeTMPyP. * *p* < 0.05 vs. control.

Previous studies have shown that peroxynitrite causes nitration of tyrosine residues on various proteins including IRS-1 and Akt [12]. This prevents tyrosine phosphorylation, resulting in inhibition of insulin-stimulated glucose uptake [12]. Having established that FeTBAP acts as a peroxynitrite decomposition catalyst, we asked whether FeTBAP would affect protein nitration levels. Treatment of C2C12 myotubes with FeTBAP for 18 h caused a decrease in nitrotyrosine levels on a prominent band at about 35 kDa (Figure 3A,B, * *p* < 0.05 versus control). FeTPPS caused a decrease in nitration at a prominent band at about 65 kDa (Figure 3C,D).

### 2.4. FeTBAP and FeTPPS Increase Akt Phosphorylation and Glucose Transport

**Figure 4.**
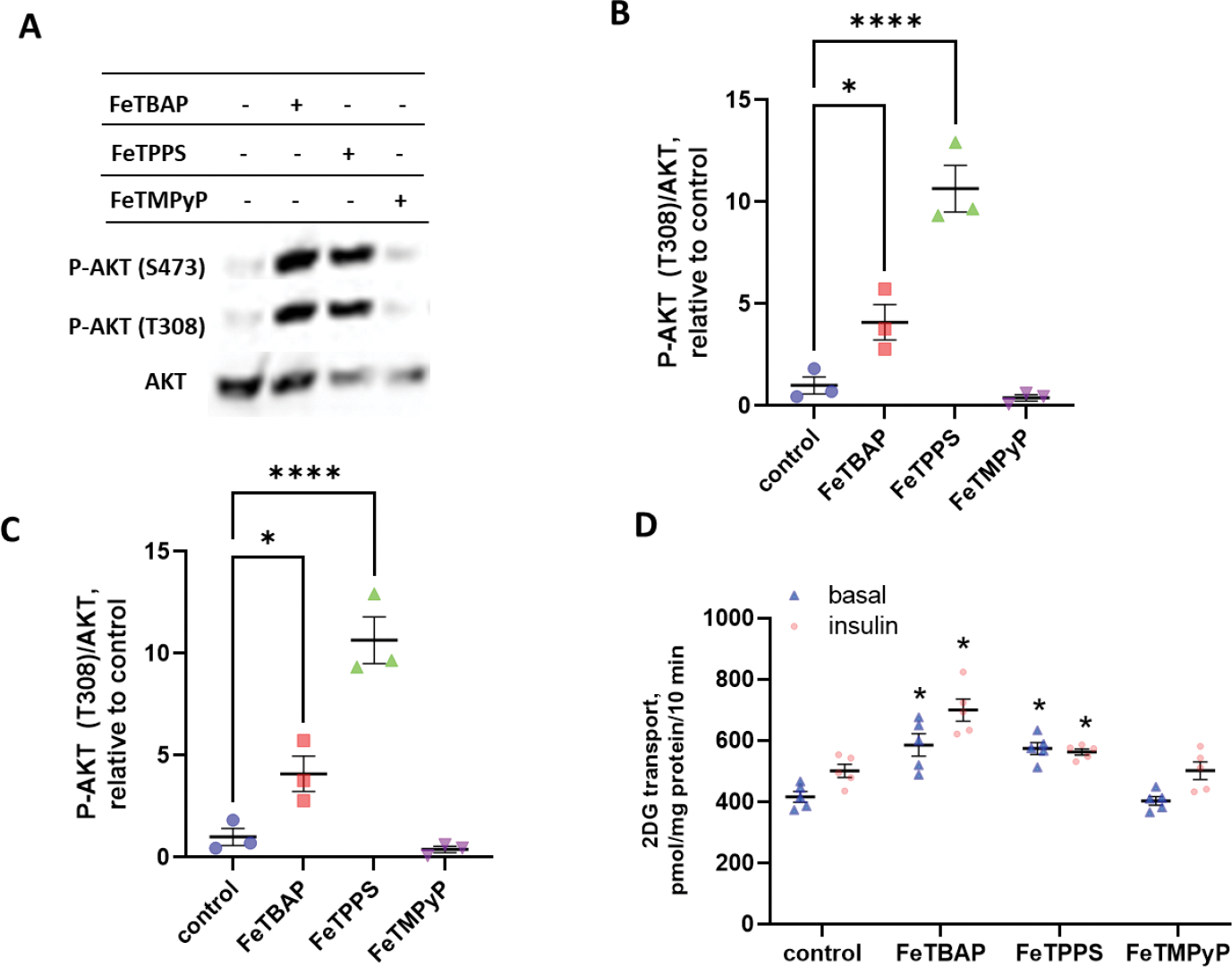
FeTBAP and FeTPPS increase insulin signaling and glucose transport in C2C12 myotubes. C2C12 myotubes were incubated in the absence or presence of 150 μM FeTBAP, FeTPPS, or FeTMPyP for 2 h. (**A**-**C**) Western blot and quantitation for for P-Akt (S473), P-Akt (Thr308), and Akt. * *p* < 0.05, **** *p* < 0.0001 versus control. (**D**) C2C12 myotubes were serum starved and incubated in the presence or absence of 150 μM FeTBAP, FeTPPS, or FeTMPyP for 2 h followed by an incubation in the presence or absence of 100 nM insulin and assay of 2-deoxyglucose (2DG) uptake. * *p* < 0.05 versus basal control.

Certain iron-containing porphyrins, such as FeTPPS, have been shown to rescue muscle cells from insulin resistance following treatment with peroxynitrite [12,13]. Further, we have recently reported that FeTBAP stimulates insulin signaling and glucose transport in skeletal muscle [16]. Thus, we asked whether Fe-porphyrin compounds, in general, have the ability to stimulate Akt phosphorylation and glucose transport. As shown in Figure 4A–C, treatment with FeTPPS or FeTBAP increased Akt phosphorylation in C2C12 myotubes (* *p* < 0.05 versus control). In contrast, FeTMPyP had no effect on Akt phosphorylation. We next determined whether the Fe-porphyrins would increase glucose uptake. Pretreatment with either FeTBAP or FeTPPS caused a significant increase in glucose transport (Figure 4D, * *p* < 0.01 versus basal control). However, pretreatment with FeTMPyP had no effect on glucose uptake.

### 2.5. FeTPPS and FeTBAP Act as Peroxidases

**Figure 5.**
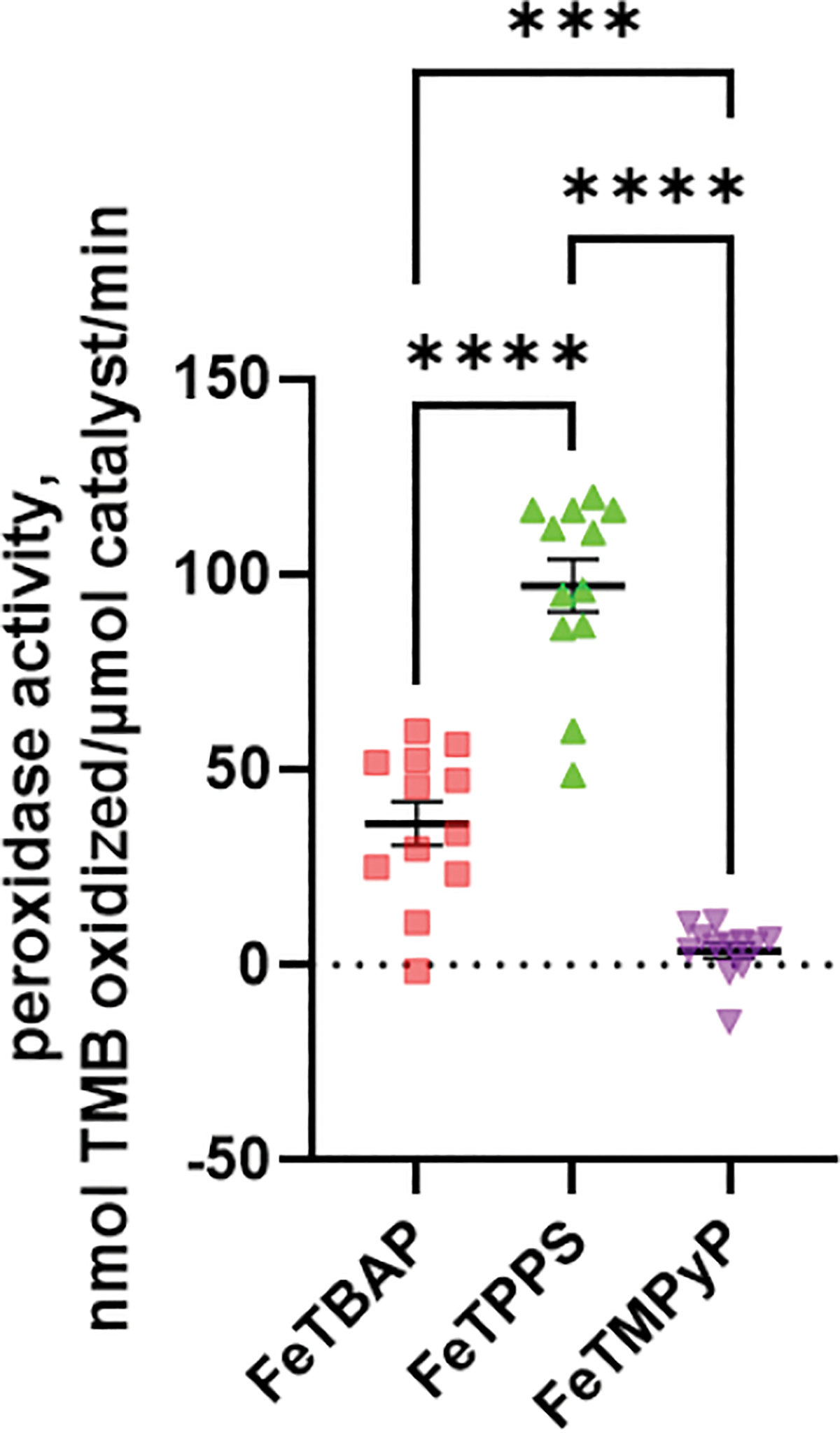
FeTPPS and FeTBAP display peroxidase activity while FeTMPyP does not. Peroxidase activity was monitored via spectrophotometric peroxidase assays containing 150 μM TMB, 150 μM FeTPPS, FeTMPyP, FeTBAP, and 150 μM hydrogen peroxide. ***p < 0.001, **** *p* < 0.0001 for comparisons indicated.

Given that FeTPPS and FeTMPyP have been characterized as peroxynitrite decomposition catalysts [9,10], we investigated whether these two porphyrins exhibited peroxidase activity as well. As shown in Figure 5, FeTBAP and FeTPPS both display peroxidase activity, while FeTMPyP does not.

## 3. Discussion

This study shows that FeTBAP has peroxynitrite scavenging abilities and decreases nitrotyrosine levels in skeletal muscle cells. Additionally, FeTPPS, a well-characterized peroxynitrite decomposition catalyst [9,19], increases insulin signaling and glucose transport in C2C12 myotubes and acts as a peroxidase. However, FeTMPyP, another well-characterized peroxynitrite decomposition catalyst [9,10], had no effect on Akt phosphorylation or glucose transport and did not exhibit peroxidase activity.

Due to its highly reactive nature, peroxynitrite contributes to disrupted cell signaling, apoptosis, and cell death [20]. Moreover, reactive nitrogen species have been implicated in various forms of pathophysiological complications and diseases including stroke, aging, cancer, and insulin resistance/diabetes [10,20]. Peroxynitrite leads to insulin resistance via the nitration of tyrosine residues on key proteins in the insulin-signaling pathway [12].

Previous work has demonstrated actions of peroxynitrite decomposition catalysts to rescue cells from insulin resistance [12,13,21]. Effects of these compounds are summarized in Table 1. Our data suggest that part of the mechanism of these compounds might be direct activation of insulin signaling and glucose transport as opposed to reversal of insulin resistance. In our previous work on FeTBAP, we found that its peroxidase activity protected skeletal muscle cells from peroxide-related insulin resisance [16]. However, FeTBAP also increased insulin signaling and glucose transport in cells that were not exposed to H_2_O_2_, even though FeTBAP did not decrease intracellular H_2_O_2_ in these cells. This suggests that some other property of FeTBAP contributes to stimulation of insulin signaling and glucose transport.

The heme breakdown product hemin has been investigated for its potential role in prevention of insulin resistance. For example, daily injections of hemin decreased whole-body glucose uptake during a hyperinsulinemic-euglycemic clamp for mice on a high fat diet [22]. Similarly, hemin injections twice a week for mice on a high fat diet decreased fasting and non-fasting plasma glucose concentrations toward the levels in chow-fed animals [23]. Likewise, hemin injections decreased fasting glucose and glucose area under the curve during an insulin tolerance test in fat-fed mice [24]. These improvements in glucoregulation were concomitant with increased insulin-stimulated phosphorylation of the insulin receptor and Akt in liver [24]. As reviewed by Schaer et al. [25], hemin regulates expression of antioxidant enzymes including heme oxygenase-1. Hemin also serves as a ligand for the nuclear receptor REV-ERB, which regulates expression of genes involved in processes including glucose metabolism [25]. Thus, hemin may act through changes in gene expression to influence glucoregulation. The current data demonstrate a role of iron porphoryins in acute regulation of insulin signaling and glucose uptake, suggesting that these compounds can act before any changes in gene expression would be realized.

Only a small number of proteins are nitrated, reflected in studies of the nitrated proteome that usually find 110 or fewer nitrated proteins [26]. A list of nitrated mouse proteins from DeepNitro [27,28], a curated database of tyrosine nitrated and S-nitrosylated sites, contains 120 proteins known to be nitrated (Table S1). None of the nitrated proteins with molecular weights approximating the molecular weights of the prominent nitrated protein bands in the current study appear to have overt roles in insulin signaling. It is possible that these bands serve of markers of overall nitration, and other nitrated proteins with roles in insulin signaling are under the threshold for detection. Future work should focus on identification of roles of nitration in insulin signaling and action. A focus in future investigation might be on proteins that can be nitrated or phosphorylated on the same tyrosine site. Nitration prevents phosphorylation of the hydroxyl group on a tyrosine [29]. Likewise, tyrosine phosphorylation impedes nitration of that tyrosine residue [29]. Thus, it will be important to understand the competitive effects of tyrosine nitration and phosphorylation in insulin signaling. Overall, our data suggest that compounds that possess both peroxidase and peroxynitrite decomposition activity are able to stimulate Akt phosphorylation and glucose transport in skeletal muscle cells. This suggests that in addition to protective effects against RNS and H_2_O_2_, these compounds can directly improve insulin signaling and glucose transport into skeletal muscle cells.

## 4. Materials and Methods

### 4.1. Materials

**Figure 6.**
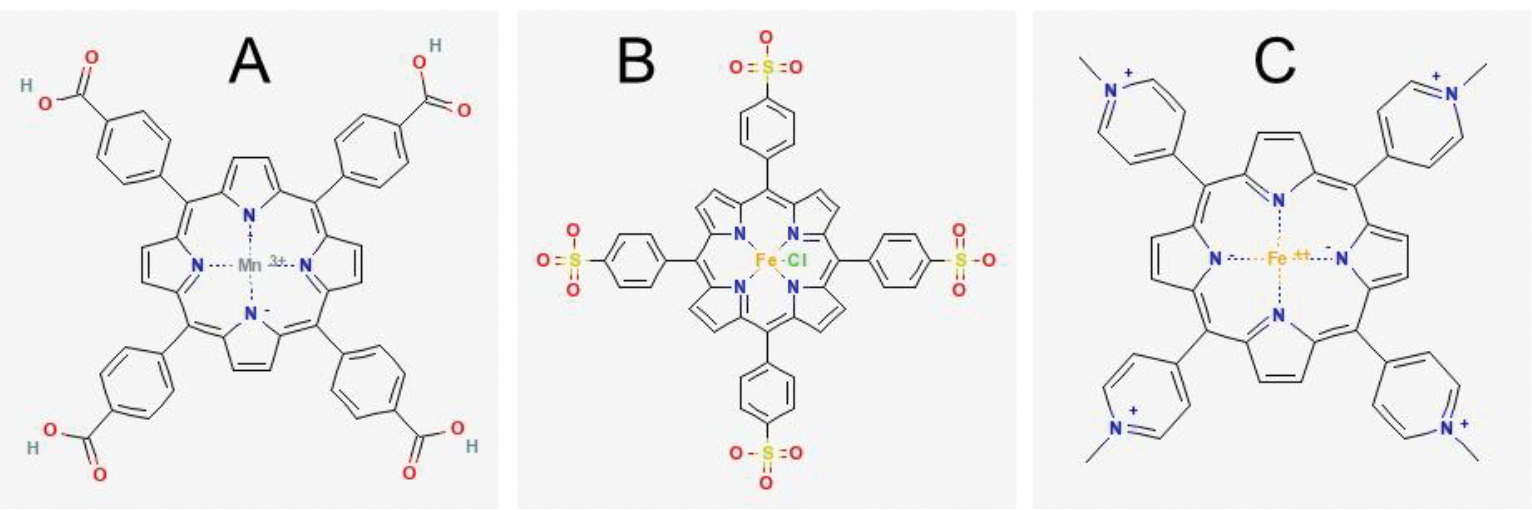
Metalloporphyrin structures. **A**. structure of MnTBAP [30]. The FeTBAP structure is the same except for iron in the spot occupied by manganese. **B**. structure of FeTPPS [31]. **C**. structure of FeTMPyP [32]. These structure images are reused without special permission needed, according to the PubChem citation guidelines on reusing the 2D or 3D structure image of a compound or substance record.

Phosphate buffered saline (PBS), trypsin-EDTA, penicillin-streptomycin, and Dulbecco’s modified Eagle’s medium (DMEM), 4,4′,4″,4‴-(porphine-5,10,15,20-tetrayl)tetrakis(benzoic acid) (TBAP), iron (II) sulfate heptahydrate, Chelex-100, (+)-6-Hydroxy-2,5,7,8-tetramethyl-chromane-2-carboxylic acid (Trolox), sodium nitrite, sodium hydrosulfite (dithionite), peroxynitrite, and primary antibody against 3-nitrotyrosine (Sigma-Aldrich Cat N5538, RRID:AB1840351, St. Louis, MO, USA) were purchased from Sigma Aldrich (St. Louis, MO, USA). Primary antibodies against phosphorylated-Akt (Ser473: Cell Signaling Technology Cat 9271, RRID:AB329825, Danvers, MA, USA), phosphorylated-Akt (Thr308: Cell Signaling Technology Cat 9275, RRID:AB329828), and pan Akt (Cell Signaling Technology Cat 9272, RRID:AB329827) were purchased from Cell Signaling Technologies (Danvers, MA, USA). S-nitrosocysteine primary antibody (Abcam Cat ab94930, RRID:AB10697568, Cambridge, MA, USA) was acquired from Abcam (Cambridge, MA, USA). D-glucose, sodium hydroxide, Pierce BCA protein assay kit, the secondary antibody goat anti-mouse conjugated to HRP, and the secondary antibody goat anti-rabbit conjugated to HRP were acquired from ThermoFisher Scientific (Rockford, IL, USA). FetalPlex was obtained from Gemini Bio-Products (Woodland, CA, USA). Horse serum was procured from Gibco Technologies (Gaithersburg, MD, USA). 4–20% SDS-PAGE gels were purchased from Expedeon (San Diego, CA, USA). Western Lighting Plus enhanced chemiluminescence reagent was obtained from Perkin Elmer (Hopkinton, MA, USA). Blotting Grade Blocker was procured from Bio-Rad Laboratories (Des Plaines, IL, USA). NADPH was purchased from Enzo (Farmingdale, NY, USA). Fe(III)5,10,15,20-tetrakis(4-sulfonatophenyl)porphyrinato chloride (FeTPPS) and Fe(III)tetrakis(1-methyl-4-pyridyl)porphyrin pentachlorideporphyrin pentachloride (FeTMPyP) were purchased from Cayman Chemical (Ann Arbor, MI, USA). FeTBAP was prepared as previously described [16]. Structures of the metalloporphyrins are shown in Figure 6.

### 4.2. Potential Role of FeTBAP as a Nitrite Reductase

In order to determine a possible role of FeTBAP as a nitrite reductase, the Soret band of 20 μM FeTBAP was monitored around 412 nm utilizing a UV-2501PC UV-Vis spectrophotometer purchased from Shimadzu Scientific (Columbia, MD, USA). FeTBAP was then reduced with one of the following reducing agents: 100 μM dithionite, 100 μM NADPH, or 100 μM Trolox. The spectrum was taken monitoring the Soret band around 412 nm. After reduction of FeTBAP, 200 μM sodium nitrite was added to the cuvette and absorbance spectra taken around 412 nm in order to monitor the effect on the Soret band of FeTBAP.

### 4.3. Cell Culture

C2C12 myoblasts were obtained from the American Type Culture Collection (Manassas, VA, USA). Cells were cultured following standard procedures [33,34] in low-glucose Dulbecco’s Modified Eagle’s medium (DMEM) without phenol red supplemented with 10% (v/v) FetalPlex (Gemini Bio, Atlanta, GA, USA) and containing penicillin-streptomycin. Cells were incubated in 5% CO_2_ at 37°C. Myoblasts were monitored and passaged every other day. Once cells reached about 70% confluence, myoblasts were differentiated into myotubes for two days in low glucose DMEM without phenol red containing 2% (*v*/*v*) horse serum and penicillin-streptomycin.

### 4.4. Effect of FeTBAP on S-Nitrosylation

To evaluate an effect of FeTBAP treatment on levels of S-nitrosylation, a spontaneous modification of cysteine side chains by NO, C2C12 myotubes of a 12-well plate were pretreated for 2 h with 150 μM FeTBAP in DMEM without phenol red. Following treatment with FeTBAP, samples were harvested in lysis buffer comprised of 50 mM HEPES pH 7.4, 150 mM NaCl, 10% (*v*/*v*) glycerol, 1.5 mM MgCl, 1 mM EDTA, 10 mM Na_4_O_7_P_2_, 100 mM NaF, 2 mM Na_3_VO4, 10 mg/mL leupeptin, 10 mg/mL aprotinin, 0.5 mg/mL pepstatin, and 1 mM phenylmethylsulfonyl fluoride. Whole cell homogenate protein content was quantified with a bicinchoninic acid (BCA) protein assay (Thermo Scientific Pierce BCA Protein Assay Kit). Western blot analysis was then performed for S-nitrosocysteine and GAPDH.

### 4.5. Western Blot Analysis

After samples were run on 4–20% gels, they were transferred to nitrocellulose membranes. Membranes were blocked in 5% (*w*/*v*) nonfat dry milk (Bio-Rad, Hercules, CA, USA) in TRIS-buffered saline (TBS) with 0.1% (*v*/*v*) Tween-20 (TBST) and then incubated with primary antibodies in 1% (*w*/*v*) nonfat dry milk. After washing with TBST, membranes were incubated with horseradish peroxidase-linked secondary antibodies. Membranes were washed with TBST and then TBS before incubation with Western Lightning Plus (PerkinElmer, Waltham, MA, USA) enhanced chemiluminescence reagent, followed by CCD imaging (iBright CL1000, ThermoFisher Scientific). The primary antibody against GAPDH (Cell Signaling Technology Cat 8884, RRID:AB11129865) was conjugated to horseradish peroxidase, so there was not a need for incubation with secondary antibodies.

### 4.6. Peroxynitrite Decomposition Activity

In order to determine a possible role of FeTBAP as a peroxynitrite decomposition catalyst, the Soret band of 20 μM FeTBAP was first monitored around 412 nm following similar studies [9]. After the spectrum of FeTBAP was obtained, 300 μM peroxynitrite was added to the 1 mL cuvette, and absorbance spectra were taken around 412 nm to monitor the effect on the Soret band of FeTBAP. The decomposition of peroxynitrite was then monitored at 302 nm. This involved a preliminary read of the absorbance of 20 μM FeTBAP at 302 nm. 300 μM peroxynitrite was then added to the 1 mL quartz cuvette, and absorbance was recorded every 10 s for 50 s at 302 nm with a Spectronic Genesys 5 (Fitchburg, WI, USA). Readings were done in 0.1 M NaOH, pH 12.4, to prevent spontaneous decomposition of peroxynitrite.

### 4.7. Nitrotyrosine Levels in C2C12 Myotubes

As our data indicated that FeTBAP can catalyze peroxynitrite decomposition, we then determined the effect of treatment with FeTBAP on nitrotyrosine levels in cultured muscle cells. C2C12 myotubes were treated for 18 h with 150 μM FeTBAP in low-glucose DMEM without phenol red. Myotubes were harvested in lysis buffer, and whole cell homogenate protein content was quantified via a BCA protein assay. Western blot analysis was then performed for 3-nitrotyrosine and GAPDH.

### 4.8. Insulin Signaling

We previously reported that FeTBAP stimulates insulin signaling and glucose transport in skeletal muscle [16]. To determine if, in general, all iron containing porphyrins increase insulin action by C2C12 myotubes, cells were pretreated with 150 μM FeTBAP, FeTPPS, or FeTMPyP for 2 h in DMEM without phenol red. Following pretreatment, myotubes were incubated for 20 min in the presence or absence of 10 nM insulin. Samples were then harvested in lysis buffer, and whole cell homogenate protein content was quantified. Western blot analysis was then performed for P-Akt (Ser473), P-Akt (Thr308), and total Akt.

### 4.9. Glucose Transport

2-DG transport assays were performed as previously described [16]. C2C12 myotubes in 24-well plates were serum starved for 3 h and pretreated for 2 h with 150 μM FeTPPS, FeTBAP, or FeTPMyP. Following pretreatment, samples were incubated in the presence or absence of 100 nM insulin. To correct for background, some wells of myotubes were incubated for 20 min in the presence of 10 μM cytochalasin B, which prevents glucose uptake via glucose transport (GLUT) proteins. All wells were then washed with HEPES-buffered saline (HBS) (20 mM HEPES sodium salt, 140 mM sodium chloride, 5 mM KCl, 2.5 mM MgSO_4_, 1 mM CaCl_2_). Myotubes were incubated for 10 min in transport medium (10 μM 2-deoxyglucose and 1 μCi/mL ^3^H-2-deoxyglucose tracer in HBS) in the presence or absence of 100 nM insulin, with background wells containing 10 μM cytochalasin B. Transport medium was removed, and cells were washed with cold 0.9% (*w*/*v*) NaCl. Samples then incubated while shaking in 0.2 N NaOH with 0.2% (*w*/*v*) SDS for 30 min. Ultima Gold scintillation fluid was added to aliquots of each sample, and vials were read on a Tri-Carb 3100 TR liquid scintillation counter (PerkinElmer, Waltham, MA, USA). Data were normalized to protein content of the samples.

### 4.10. Peroxidase Activity

Peroxidase activity of FeTBAP, FeTPPS, and FeTMPyP was monitored utilizing TMB. 150 μM FeTBAP, FeTPPS, and FeTMPyP were reacted with 150 μM hydrogen peroxide and 150 μM TMB in a 100 mM sodium phosphate buffer pH 7.4. Absorbance was monitored every minute for 10 min at 653 nm. Oxidation of TMB was quantified using the extinction coefficient: 39 mM^−1^cm^−1^ [35].

### 4.11. Statistics.

Comparisons of two means were done with Student’s t tests. Time course data were analyzed with a repeated measures ANOVA followed by post hoc comparisons of the experimental group versus the control group for each time point. Multiple group comparisons were performed by ANOVA followed by Dunnett post hoc tests when there was a control group or LSD post hoc tests to compare all groups.

### 4.12. Nitrated Mouse Proteins

A list of known nitrated proteins was obtained from the DeepNitro [27,28] database of nitrated and S-nitrosylated proteins from humans, mice, yeast, *Arabidopsis*, and *Drosophila*. These 761 proteins were cross-referenced against the entire mouse UniProt database [36,37] to obtain a group of 120 mouse proteins known to be tyrosine-nitrated. Molecular weights for these proteins were obtained from UniProt.

## Supplementary Material

Table S1 Nitrated mouse protein

## Figures and Tables

**Table 1.** Effects of Fe-porphyrin treatment in insulin resistant or diabetic mice.

Fe-Porphyrin	Author(s)	Conditions	Results
FeTPPS	Zhou et al. [12]	Insulin resistant high fat diet-fed (HFD) mice	Administration of FeTPPS improved muscle insulin signaling and whole body insulin sensitivity
FeTPPS	Duplain et al. [13]	Insulin resistant high fat diet-fed mice	FeTPPS treatment restored insulin signaling and glucose uptake. Diminished HFD-induced insulin resistance in mice
FeTMPS	Drel et al. [21]	Streptozotocin induced type 1 diabetic mice	Alleviated various symptoms associated with diabetic neuropathy including manifest motor and sensory nerve conduction velocity deficits

## Data Availability

Data is contained within the article or supplementary material.

## References

[1] Gladwin MT; Kim-Shapiro DB The functional nitrite reductase activity of the heme-globins. Blood 2008, 112, 2636–2647. 10.1182/blood-2008-01-115261. 18596228 PMC2556603

[2] Shiva S; Huang Z; Grubina R; Sun J; Ringwood L; MacArthur PH; Xu X; Murphy E; Darley-Usmar V; Gladwin MT Deoxymyoglobin Is a Nitrite Reductase That Generates Nitric Oxide and Regulates Mitochondrial Respiration. Circ. Res. 2007, 100, 654–661. 10.1161/01.RES.0000260171.52224.6b. 17293481

[3] Rassaf T; Flögel U; Drexhage C; Hendgen-Cotta U; Kelm M; Schrader J Nitrite Reductase Function of Deoxymyoglobin. Circ. Res. 2007, 100, 1749–1754, doi:doi:10.1161/CIRCRESAHA.107.152488. 17495223

[4] Carvalho-Filho MA; Ueno M; Hirabara SM; Seabra AB; Carvalheira JB; de Oliveira MG; Velloso LA; Curi R; Saad MJ S-nitrosation of the insulin receptor, insulin receptor substrate 1, and protein kinase B/Akt: A novel mechanism of insulin resistance. Diabetes 2005, 54, 959–967. 15793233 10.2337/diabetes.54.4.959

[5] Badal S; Brown PD; Ragoobirsingh D Nitric oxide agents impair insulin-mediated signal transduction in rat skeletal muscle. BMC Biochem. 2006, 7, 17. 10.1186/1471-2091-7-17. 16729893 PMC1524779

[6] Stamler JS; Meissner G Physiology of nitric oxide in skeletal muscle. Physiol. Rev. 2001, 81, 209–237. 10.1152/physrev.2001.81.1.209. 11152758

[7] Lane P; Gross SS Cell signaling by nitric oxide. Semin. Nephrol. 1999, 19, 215–229. 10226328

[8] Beckman JS; Beckman TW; Chen J; Marshall PA; Freeman BA Apparent hydroxyl radical production by peroxynitrite: Implications for endothelial injury from nitric oxide and superoxide. Proc. Natl. Acad. Sci. USA 1990, 87, 1620–1624. 2154753 10.1073/pnas.87.4.1620 PMC53527

[9] Stern MK; Jensen MP; Kramer K Peroxynitrite Decomposition Catalysts. J. Am. Chem. Soc. 1996, 118, 8735–8736. 10.1021/ja961279f.

[10] Salvemini D; Wang ZQ; Stern MK; Currie MG; Misko TP Peroxynitrite decomposition catalysts: Therapeutics for peroxynitrite-mediated pathology. Proc. Natl. Acad. Sci. USA 1998, 95, 2659–2663. 9482943 10.1073/pnas.95.5.2659 PMC19452

[11] Radi R Oxygen radicals, nitric oxide, and peroxynitrite: Redox pathways in molecular medicine. Proc. Natl. Acad. Sci. USA 2018, 115, 5839. 10.1073/pnas.1804932115. 29802228 PMC6003358

[12] Zhou J; Huang K Peroxynitrite mediates muscle insulin resistance in mice via nitration of IRbeta/IRS-1 and Akt. Toxicol. Appl. Pharmacol. 2009, 241, 101–110. 10.1016/j.taap.2009.08.005. 19682478

[13] Duplain H; Sartori C; Dessen P; Jayet P-Y; Schwab M; Bloch J; Nicod P; Scherrer U Stimulation of peroxynitrite catalysis improves insulin sensitivity in high fat diet-fed mice. J. Physiol. 2008, 586, 4011–4016. 10.1113/jphysiol.2008.154302. 18591189 PMC2538923

[14] Stamler JS; Singel DJ; Loscalzo J Biochemistry of nitric oxide and its redox-activated forms. Science 1992, 258, 1898–1902. 1281928 10.1126/science.1281928

[15] Stamler JS Redox signaling: Nitrosylation and related target interactions of nitric oxide. Cell 1994, 78, 931–936. 7923362 10.1016/0092-8674(94)90269-0

[16] Eccardt AM; Pelzel RJ; Mattathil L; Moon YA; Mannino MH; Janowiak BE; Fisher JS A peroxidase mimetic protects skeletal muscle cells from peroxide challenge and stimulates insulin signaling. Am. J. Physiol. Cell Physiol. 2020, 318, C1214–C1225,. 10.1152/ajpcell.00167.2019. 32348172 PMC7311744

[17] Bryan NS; Grisham MB Methods to detect nitric oxide and its metabolites in biological samples. Free Radic. Biol. Med. 2007, 43, 645–657. 10.1016/j.freeradbiomed.2007.04.026. 17664129 PMC2041919

[18] Hare JM; Beigi F; Tziomalos K Chapter Twenty-One-Nitric Oxide and Cardiobiology-Methods for Intact Hearts and Isolated Myocytes. In Methods in Enzymology; Cadenas E, Packer L, Eds.; Academic Press: San Diego, CA, USA, 2008; Volume 441, pp. 369–392. 18554546 10.1016/S0076-6879(08)01221-4

[19] Lauzier B; Sicard P; Bouchot O; Delemasure S; Moreau D; Vergely C; Rochette L A peroxynitrite decomposition catalyst: FeTPPS confers cardioprotection during reperfusion after cardioplegic arrest in a working isolated rat heart model. Fundam. Clin. Pharmacol. 2007, 21, 173–180. 10.1111/j.1472-8206.2007.00467.x. 17391290

[20] Stadler K Peroxynitrite-driven mechanisms in diabetes and insulin resistance - the latest advances. Curr. Med. Chem. 2011, 18, 280–290. 21110800 10.2174/092986711794088317 PMC4191845

[21] Drel VR; Pacher P; Vareniuk I; Pavlov IA; Ilnytska O; Lyzogubov VV; Bell SR; Groves JT; Obrosova IG Evaluation of the peroxynitrite decomposition catalyst Fe(III) tetra-mesitylporphyrin octasulfonate on peripheral neuropathy in a mouse model of type 1 diabetes. Int. J. Mol. Med. 2007, 20, 783–792. 17982684 PMC2527588

[22] Ju TJ; Kwon WY; Kim YW; Kim JY; Kim YD; Lee IK; Park SY Hemin improves insulin sensitivity in skeletal muscle in high fat-fed mice. J. Pharm. Sci. 2014, 126, 115–125. 10.1254/jphs.14003fp. 25341564

[23] Szkudelski T; Dłużewicz K; Sadoch J; Szkudelska K Effects of the activation of heme oxygenase-1 on hormonal and metabolic changes in rats fed a high-fat diet. Biomed. Pharm. 2017, 87, 375–380. 10.1016/j.biopha.2016.12.060. 28068626

[24] Luan Y; Zhang F; Cheng Y; Liu J; Huang R; Yan M; Wang Y; He Z; Lai H; Wang H; Hemin Improves Insulin Sensitivity and Lipid Metabolism in Cultured Hepatocytes and Mice Fed a High-Fat Diet. Nutrients 2017, 9. 10.3390/nu9080805. PMC5579599 28933767

[25] Schaer DJ; Buehler PW; Alayash AI; Belcher JD; Vercellotti GM Hemolysis and free hemoglobin revisited: Exploring hemoglobin and hemin scavengers as a novel class of therapeutic proteins. Blood 2013, 121, 1276–1284. 10.1182/blood-2012-11-451229. 23264591 PMC3578950

[26] Batthyány C; Bartesaghi S; Mastrogiovanni M; Lima A; Demicheli V; Radi R Tyrosine-Nitrated Proteins: Proteomic and Bioanalytical Aspects. Antioxid. Redox Signal 2017, 26, 313–328. 10.1089/ars.2016.6787. 27324931 PMC5326983

[27] DeepNitro. Available online: http://deepnitro.renlab.org/ (accessed on 15 July 2022).

[28] Xie Y; Luo X; Li Y; Chen L; Ma W; Huang J; Cui J; Zhao Y; Xue Y; Zuo Z; DeepNitro: Prediction of Protein Nitration and Nitrosylation Sites by Deep Learning. Genom. Proteom. Bioinform. 2018, 16, 294–306. 10.1016/j.gpb.2018.04.007. PMC6205083 30268931

[29] Abello N; Kerstjens HA; Postma DS; Bischoff R Protein tyrosine nitration: Selectivity, physicochemical and biological consequences, denitration, and proteomics methods for the identification of tyrosine-nitrated proteins. J. Proteome Res. 2009, 8, 3222–3238. 10.1021/pr900039c. 19415921

[30] National Library of Medicine (US). National Center for Biotechnology Information, PubChem Compound Summary for CID 6610341, Mntbap. Available online: https://pubchem.ncbi.nlm.nih.gov/compound/6610341#section=2D-Structure (accessed on 15 July 2022).

[31] National Library of Medicine (US). National Center for Biotechnology Information, PubChem Substance Record for SID 26758697. Available online: https://pubchem.ncbi.nlm.nih.gov/substance/26758697#section=2D-Structure (accessed on 15 July 2022).

[32] National Library of Medicine (US). National Center for Biotechnology Information, PubChem Compound Summary for CID 16760420, FeTMPyP. Available online: https://pubchem.ncbi.nlm.nih.gov/compound/16760420#section=2D-Structure (accessed on 15 July 2022).

[33] Andrisse S; Patel GD; Chen JE; Webber AM; Spears LD; Koehler RM; Robinson-Hill RM; Ching JK; Jeong I; Fisher JS ATM and GLUT1-S490 Phosphorylation Regulate GLUT1 Mediated Transport in Skeletal Muscle. PLoS ONE 2013, 8, e66027. 10.1371/journal.pone.0066027. 23776597 PMC3679034

[34] Eccardt AM; Bell TP; Mattathil L; Prasad R; Kelly SC; Fisher JS Trans-Plasma Membrane Electron Transport and Ascorbate Efflux by Skeletal Muscle. Antioxidants 2017, 6, 89. 10.3390/antiox6040089. 29120354 PMC5745499

[35] Mirazizi F; Bahrami A; Haghbeen K; Shahbani Zahiri H; Bakavoli M; Legge RL Rapid and direct spectrophotometric method for kinetics studies and routine assay of peroxidase based on aniline diazo substrates. J. Enzym. Inhib. Med. Chem. 2016, 31, 1162–1169. 10.3109/14756366.2015.1103234. 26526616

[36] UniProt: The universal protein knowledgebase in 2021. Nucleic Acids Res 2021, 49, D480–D489,. 10.1093/nar/gkaa1100. 33237286 PMC7778908

[37] UniProt. Available online: https://www.uniprot.org/ (accessed on 15 July 2022).

